# Mechanisms of mitral valve development and disease

**DOI:** 10.3389/fcvm.2026.1773671

**Published:** 2026-02-04

**Authors:** Enshi Wang, Bin Zhou

**Affiliations:** 1 Department of Pediatrics, The University of Chicago, Chicago, IL, United States; 2 Departmens of Genetics, Pediatrics, and Medicine, Albert Einstein College of Medicine, New York, NY, United States

**Keywords:** congenital mitral stenosis, epigenetic, genetic, mitral valve, myxomatous mitral valve prolapse, rheumatic mitral stenosis

## Abstract

The mitral valve apparatus comprises the annulus, valve leaflets, chordae tendineae, and papillary muscles, forming an integrated biomechanical unit essential for unidirectional blood flow. The leaflets and chordae are primarily derived from endocardial cells, and damage to these structures results in either mitral stenosis or mitral regurgitation, depending on the underlying pathology. This review compares three major mitral valve diseases, rheumatic mitral stenosis, congenital mitral stenosis, and myxomatous mitral valve prolapse, to highlight their distinct etiologies, molecular mechanisms, and structural endpoints. Rheumatic mitral stenosis is an acquired immune-mediated disease triggered by Group A streptococcal infection, in which molecular mimicry leads to autoantibody formation and chronic inflammation. Immune-cell infiltration and cytokine release drive the progression of leaflet fibrosis, commissural fusion, calcification, and pronounced chordal shortening, ultimately culminating in fixed obstruction. Large-scale genetic studies have not identified strong causal genes, instead revealing associations with immune-related risk loci, while valve-specific epigenetic mechanisms are poorly explored. Congenital mitral stenosis arises from developmental abnormalities of the mitral valve complex during embryogenesis and is classified into four anatomical subtypes. Due to its low incidence, the condition remains the least studied at the molecular and genetic levels. In contrast, myxomatous mitral valve prolapse is a degenerative, polygenic disorder driven by aberrant TGF*β*-dependent endothelial-to-mesenchymal transformation, valve interstitial cell activation, and extracellular matrix remodeling. Genetic studies have identified multiple causal genes, including *FLNA*, *DCHS1*, *DZIP1*, and *TNS1*, underscoring its mechano-genetic origin. Despite their distinct causes, immune-mediated, developmental, and degenerative/genetic, all three diseases converge on progressive structural failure of the MV apparatus. Notably, pathological remodeling of the chordae plays a decisive role in disease progression and the need for surgical intervention. A deeper understanding of both shared and disease-specific mechanisms, particularly valve- and chordae-specific molecular regulation, is essential to advance translational research in mitral valve disease.

## 1 Introduction

Mitral valve (MV) diseases remain a major cause of cardiovascular morbidity and represent a leading indication for cardiac surgery worldwide (1). Although these disorders share a common anatomical target, the mitral valve apparatus, they arise from fundamentally distinct etiologies and follow divergent pathogenic trajectories. Understanding both their structural consequences and disease-specific mechanisms is essential for advancing diagnosis and therapy. In this review, we focus on three major categories of MV disease. Mitral stenosis (MS) is represented primarily by rheumatic mitral stenosis (RMS), an acquired immune-mediated disorder following Group A streptococcal (GAS) infection, and congenital mitral stenosis (CMS), a rare form of congenital heart disease resulting from developmental malformations established during embryogenesis, and mitral regurgitation (MR) caused by myxomatous mitral valve prolapse (MMVP) represents a progressive degenerative condition characterized by extracellular matrix (ECM) remodeling, valve leaflet redundancy, and strong genetic predisposition. We begin by outlining the developmental origins and structural organization of the MV apparatus, then integrate current knowledge of molecular signaling pathways, genetic determinants, epigenetic regulation, and experimental models that underlie RMS, CMS, and MMVP. Our goal is to highlight disease-specific pathways that shape MV pathology, with particular emphasis on the subvalvular apparatus, especially the chordae tendineae (CT), as its pathological remodeling is a decisive factor driving clinical deterioration and the need for surgical intervention. A deeper understanding of these mechanisms will be critical for improving diagnostic precision and developing disease-modifying therapies for MV disease.

## 2 Mitral valve anatomy

The MV apparatus consists of the valve leaflets, CT, papillary muscles (PMs), and the mitral annulus, which together maintain unidirectional blood flow between the left atrium (LA) and left ventricle (LV) (2–4). Anatomically, the MV contains anterior (aortic) and posterior (mural) leaflets. The anterior leaflet has a larger surface area, whereas the posterior leaflet occupies a greater portion of the annular circumference (5). CTs are categorized into primary, secondary, and tertiary types (6). Primary chordae insert into the free margins of the leaflet essential for leaflet coaptation. The shortening or fusion of the primary chordae restricts leaflet excursion, which is a major mechanism of congenital or acquired MS (5, 7). Secondary chordae insert onto the ventricular surface of the leaflets, where they help in maintaining left ventricular geometry and distribute mechanical load across the leaflet-ventricle interface (6, 7). Tertiary chordae connect the posterior leaflet directly to the ventricular wall, possibly serving as stabilizers during systolic tension, but their biomechanical functions remain poorly defined. Two PMs, anterolateral and posteromedial, anchor the CTs to the left ventricular myocardium. The anterolateral PM (APM) typically originates as a single muscle from the anterior and lateral ventricular walls, whereas the posteromedial PM (PPM) comprises multiple heads arising from the junction of the interventricular septum and the posterior wall. CTs from each PM attach to the corresponding half of each leaflet (2–7). Anatomical studies demonstrate substantial variability: 4–22 chordae may arise from the anterolateral PM (inserting as 14–72 chordal attachments), and 2–18 may arise from the posteromedial PM (inserting as 12–80 attachments) (8). This broad range of inserts reflects the complex mechanical demand of load sharing, redundancy, and protection against chordal rupture.

## 3 Mitral valve histology

**Figure 1:**
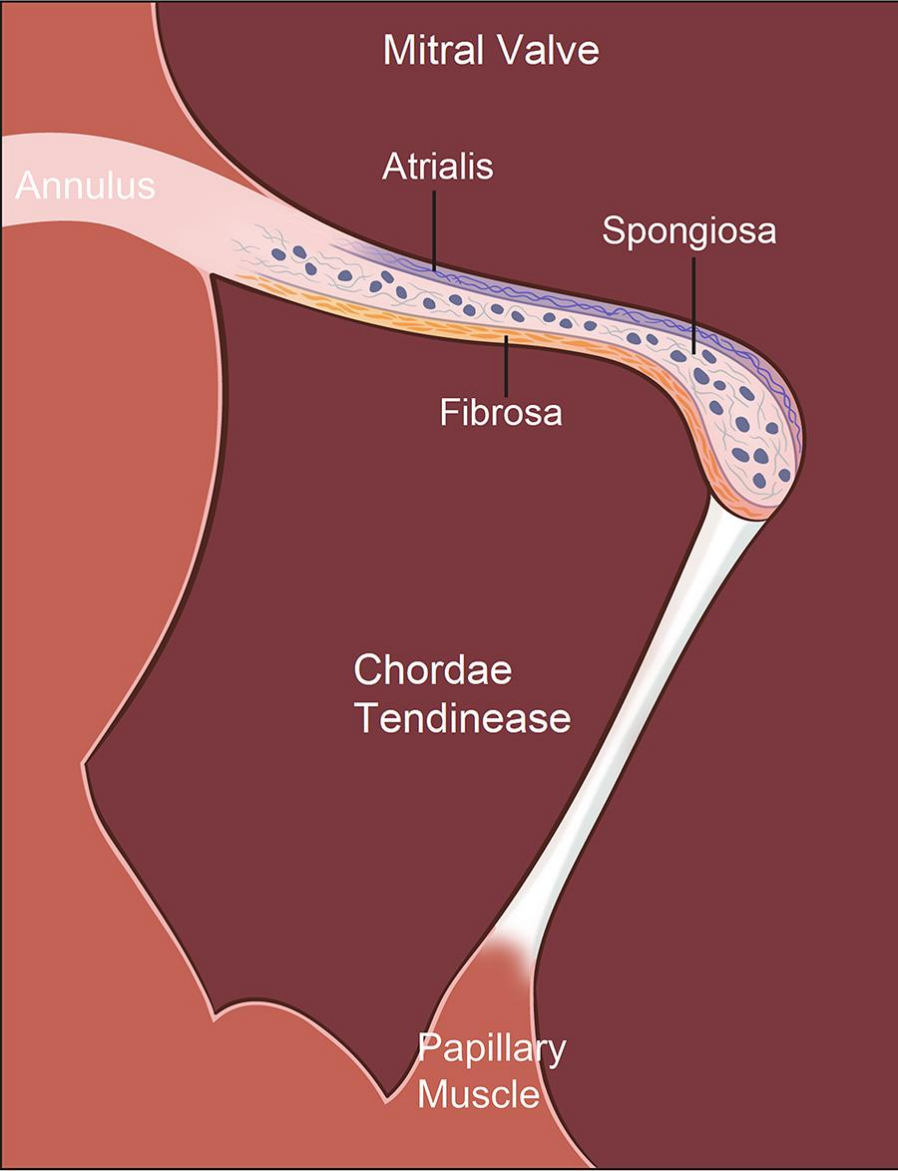
Histological organization of the mitral valve (MV) apparatus. The MV apparatus consists of the annulus, valve leaflets, chordae tendineae (CTs), and papillary muscles (PMs), functioning together as an integrated mechanical unit. Mature MV leaflets display a characteristic trilaminar architecture: (1) the fibrosa, a dense, circumferentially aligned layer rich in type I and III collagen that provides tensile strength and structural rigidity required for systolic coaptation; (2) the spongiosa, a compliant, proteoglycan- and glycosaminoglycan (GAG)–rich layer containing loosely organized collagen that absorbs shear forces and buffers mechanical transitions between the fibrosa and atrialis; and (3) the atrialis, an elastin-enriched layer with radially oriented elastic fibers that allows leaflet extensibility during ventricular pressurization and rapid recoil during diastole, supporting energy-efficient leaflet motion. These 3 layers enable the MV to withstand continuous cyclic loading while maintaining durability and flexibility across the cardiac cycle.

Mature MV leaflets are trilaminar, consisting of the fibrosa, spongiosa, and atrialis layers, characterized by distinct ECM components (9–11) (Figure 1). Fibrosa (load-bearing layer) is made of dense circumferential type I and III collagen, providing tensile strength and stiffness, ensuring firm coaptation during systole (12, 13). Atrialis (elastic layer) contains radially oriented elastic fibers, allowing leaflet extensibility as ventricular pressure rises and recoils during diastole, supporting rapid and energy-efficient leaflet motion (14). Spongiosa (cushioning layer) is composed of proteoglycans, glycosaminoglycans (GAGs), and interspersed collagen fibers, which dissipate shear forces and buffer adjacent layers during cyclic loading (9–11). Its composition and hydration also permit leaflet flexibility, essential for smooth coaptation. MV is composed of specialized cell populations that support its structure and biomechanics. Valve endothelial cells (VECs) maintain anti-thrombotic surfaces, respond to shear stress, and secrete regulatory factors that modulate inflammation and matrix turnover. Valve interstitial cells (VICs), fibroblast-like and highly plastic, synthesize collagen, elastin, and proteoglycans. Mechanical loading activates VICs, shifting them toward myofibroblast-like phenotypes, which is a main mechanism of ECM remodeling. Regional heterogeneity in VIC activation is increasingly recognized as fundamental to MV disease progression, including fibrosis, calcification, leaflet stiffness, and myxomatous change (14).

The MV apparatus handles mechanical forces through a highly integrated structural network. The trilaminar leaflet architecture compartmentalizes biomechanical function, *i.e*., the fibrosa bears tensile load and provides stiffness, the atrialis enables rapid deformation and elastic recoil, and the spongiosa absorbs shear forces while reducing friction between layers. CTs act jointly as non-linear spring systems that prevent leaflet prolapse during systole, buffer abrupt changes in ventricular pressure, and guide geometric leaflet coaptation during systole. The fibrosa of CTs was comprised of close-packed collagen fibrils intermixed with elastic fibers, both of which were closely associated with proteoglycans (15). CTs connect to the PPM are fewer in number, but significantly longer and stiffer than CTs connect to the APM, which are consistent with their higher collagen core ratio and larger collagen fibril density attachments to the PPM (16). PM contraction is synchronized with ventricular systole to maintain appropriate chordal tension that limits leaflet billowing and reduces the shear stress on the fibrosa. No significant mechanical or microstructural differences are present along the circumferential and longitudinal directions of APM and PPM (16). When any component of the MV apparatus is disrupted, such as leaflet stiffening or myxomatous change, CT shortening or elongation, fusion or rupture, or PM malposition, mechanical stress is redistributed to the remaining structures. Over time, this maladaptive overload promotes progressive MV dysfunction and eventually clinically significant MS or MR.

## 4 Developmental origins of the mitral valve

MV develops from the embryonic endocardial cushions located within the atrioventricular canal through two major morphogenetic processes: endocardial-to-mesenchymal transformation (EndoMT) and valve elongation (9, 17). In mice, EndoMT takes place from embryonic day (E) 9.5–11.5, when a subset of endocardial cells (ECCs) lining the cushions delaminate, migrate into the cardiac jelly, and form the mesenchymal core of the MV primordium (18–20). After E11.5, these early mesenchymal cells differentiate into the valve interstitial tissue, which synthesizes ECM proteins (9, 21, 22), while the surface ECCs continue to proliferate. Together, they remodel the leaflets by driving leaflet elongation, ECM deposition, and stratification. The leaflet remodeling is most prominent during late gestation and continues into the postnatal period (23). Both anterior and posterior leaflets originate from the ECCs-derived mesenchyme through EndoMT. In addition, the development of the posterior leaflet apparatus uniquely receives contributions from the epicardial-derived cells (EPDCs) (20, 22). EPDCs migrate from the atrioventricular sulcus into the myocardium and contribute to the annulus fibrosus under BMP signaling (24). This annular tissue is critical for electrically and mechanically separating atrial from ventricular myocardium (25, 26). EPDCs also populate the lateral endocardial cushion, which gives rise to the posterior leaflet. Remarkably, up to 50% of posterior leaflet mesenchymal cells derive from EPDCs (26, 27). Consequently, disrupted migration or differentiation of EPDCs can lead to structural abnormalities of the posterior leaflet and annulus (22, 27).

While leaflet development has been studied extensively (28–31), the formation of supporting CTs is understudied, despite the critical role of CTs in supporting the leaflet function, which is affected in congenital and rheumatic MS (32–34). Initial evidence suggests that, like the leaflets, CTs arise from ECC-derived mesenchyme (35, 36). Our unpublished mouse studies indicate that the morphogenesis of CTs undergoes three distinct phases. At first, primitive CTs begin to emerge around E12.5–E13.5 as mesenchymal ridges connecting the leaflet's tip and the trabeculated myocardium as early tethering structures that will later insert into the primitive PMs developed from the trabeculae. From E14.5 to E18.5 or before birth, primitive CTs undergo differentiation and elongation in concert with the leaflet stratification and PM maturation. During this remodeling period, the chordae acquire significant ECM deposition and organization, and the interface between chordae and maturing PMs becomes clearly defined. Perinatally or between E18.5 to postnatal day (P) 7, CTs transition from immature mesenchymal cords to collagen-rich tendon-like structures that are ready for supporting the leaflet function as the heart becomes fully functional after birth. Molecular characteristics of the P7 CTs include the strong expression of mesenchymal and tendon-associated markers, such as EMP-1, collagen VI, and SCX (37), reflecting progressive matrix deposition and tensile specialization. In parallel, PMs differentiate from trabeculated ventricular myocardium and condense into compact muscle bundles at E14.5, forming the anchoring base required for controlling systolic tension. Proper spatial alignment between elongating CTs and maturing PMs is essential for leaflet tethering and efficient valve mechanics.

Despite these insights, fundamental knowledge gaps remain in our understanding of CT morphogenesis. Future investigations are critically needed to identify the molecular mechanisms controlling the cell events required for the proper formation of CTs. Specifically, (1) How do CTs originate, elongate, and mature during development? (2) The differentiation trajectory of ECC-derived progenitors that populate CTs. (3) How do the ECC-derived cells interact with emerging PM cardiomyocytes? And (4) How is CT development genetically and epigenetically perturbed during MS formation? Answering these questions will provide essential mechanistic insight into MV developmental biology and the pathogenesis of MS.

## 5 Mitral stenosis

MS can be broadly classified into acquired and congenital forms. The most common acquired type is RMS, which develops as a chronic sequela of rheumatic heart valve disease (RHVD). Globally, RHVD affects approximately 41 million individuals and remains the leading cause of primary valve heart disease (VHD) and VHD-related mortality worldwide (1, 38).

### 5.1 Rheumatic mitral stenosis

In RMS, pathological remodeling occurs at both the valve and subvalvular apparatus. The mitral leaflets become thickened, fibrotic, calcified, and often show myxomatous degeneration along with commissural fusion. These changes reduce leaflet flexibility and impair opening during diastole. Simultaneously, CTs become shortened, thickened, fibrotic, and retracted, drawing the leaflet edges toward the PMs and further restricting leaflet excursion. Together, these alterations markedly decrease the effective mitral orifice area, causing MS (39, 40). The narrowed orifice obstructs emptying of the LA, resulting in rising LA pressure and progressive LA dilation. Elevated LA pressure predisposes to atrial fibrillation and promotes atrial thrombus formation. Retrograde transmission of pressure into the pulmonary circulation can lead to pulmonary congestion, pulmonary edema, and eventually pulmonary hypertension, which accelerates the clinical progression of MS and contributes to right-sided heart failure.

Acute rheumatic fever (ARF) is an autoimmune response triggered by infection with GAS. The inflammatory damage to the MV apparatus is primarily mediated by molecular mimicry, as GAS expresses several antigens, most notably the M protein that shares peptide sequence similarity with human cardiac proteins (41–43). Kaplan first reported this phenomenon in 1960 by showing that antibodies generated against GAS cross-reacted with human heart tissue (41, 44, 45). Among the cross-reactive targets, sarcomeric myosin received early attention because M protein shares sequence homology with cardiac myosin heavy chains (MYH6/α-MHC, MYH7/β-MHC) (46–49), which are abundantly expressed in contractile cardiomyocytes (50, 51).

However, RMS manifests most prominently in the mitral leaflets, CTs, and subvalvular apparatus, which are not known to have a significant contribution from the cardiac muscle tissues. This raises an important question: whether the MV cells express myosin. During embryonic development, ECC-derived mesenchymal cells transiently express sarcomeric myosins as part of EndoMT and early differentiation (36). In postnatal and adult valves, mature VICs generally lack high-level expression of cardiac myosins. Instead, subsets of VICs and fibroblast-like cells express non-muscle myosins, e.g., MYH9 and smooth muscle–type myosins, e.g., MYH11 (52). Such expression is particularly enriched in the subvalvular apparatus, including CTs and annulus (9). Although it is not clear whether these non-muscle myosins can be an antibody target, as the cardiac myosins, inflammation, mechanical stress, or cytokine signaling can activate otherwise quiescent VICs (qVICs) into myofibroblasts, which upregulate non-muscle myosin and contribute to ECM remodeling (53). Indeed, in the healthy valves, most VICs remain quiescent, maintaining basal ECM turnover, whereas in RMS, the number of activated VICs (aVICs) in the leaflets increases dramatically, promoting maladaptive matrix remodeling, fibrosis, and calcification (53). Therefore, in addition to the M protein-myosin molecular mimicry as the classical paradigm of RMS, multiple structural components of the MV can be targeted by the autoimmune response. They include the valve endothelium (54–56), laminin (48, 54, 56), basement membrane (laminin–collagen IV complex) (45, 49, 54, 56), Glycoproteins and N-acetyl-β-D-glucosamine (GlcNAc)-containing ECM molecules (57), and collagen type I and IV (58–60).

#### 5.1.1 Endothelial damage and inflammatory activation

**Figure 2:**
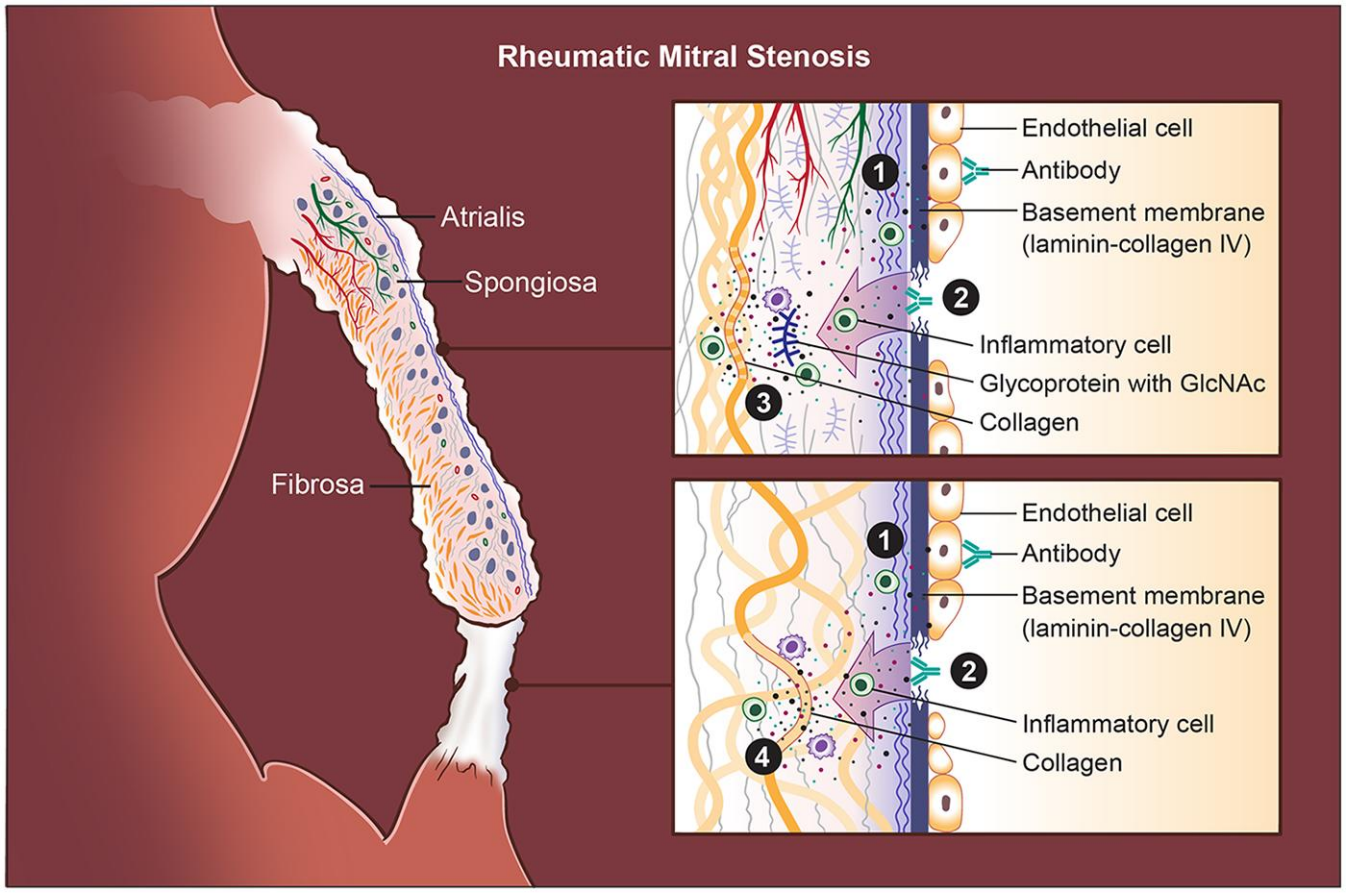
Pathogenic cascade in rheumatic mitral stenosis (RMS). Rheumatic mitral stenosis (RMS) is driven by an autoimmune response initiated by antigenic mimicry between Group A streptococcal (GAS) antigens and structural components of the mitral valve (MV). (1) Endothelial injury is the initiating event: cross-reactive antibodies bind to valve endothelial cells, disrupting intercellular junctions and causing endothelial loosening, damage, and increased permeability. Activated endothelial cells become adhesive and pro-inflammatory, permitting infiltration of T lymphocytes and other immune cells into the leaflet. (2) Exposure and immune targeting of the basement membrane occur as endothelial disruption uncovers laminin- and collagen IV–rich layers; antibodies directed against these components further amplify tissue injury. (3) Stromal autoimmunity and extracellular matrix (ECM) degradation follow as inflammatory cytokines and antibodies against GlcNAc-containing glycoproteins and collagen I penetrate the spongiosa, promoting matrix breakdown and remodeling. Immune complex deposition concurrently induces aberrant neovascularization and lymphangiogenesis within the leaflet, sustaining chronic inflammation. Chordae tendineae (CT) undergo a parallel pattern of immune-mediated injury. Unlike leaflets, where glycoproteins are abundant, the CT core is densely collagenous; thus, (4) autoantibodies predominantly target collagen I within the chordae, leading to accentuated collagen injury, fibrosis, and progressive chordal shortening. Finally, these immune-driven injuries produce leaflet thickening, stromal fibrosis, commissural fusion, and CT shortening, eventually resulting in fixed obstruction of the mitral orifice and the hemodynamic consequences characteristic of RMS.

Autoantibodies from RMS patients bind to endothelial antigens and trigger marked endothelial activation (55) (Figure 2). Activated VECs produce increased adhesion and ECM-related proteins, including ICAM-1, VCAM-1, E-selectin, laminin B, and vimentin, along with pro-inflammatory cytokines such as TNF-α, IL-6, IL-8, IFN-γ, and IL-1β. The activation of VECs enhances leukocyte adhesion and transmigration, driving inflammatory cell infiltration into the leaflet tissue, resulting in cytokines and matrix-remodeling environments that initiate progressive ECM deposition, leaflet thickening, and fibrosis.

#### 5.1.2 Laminin and basement membrane as autoantigenic targets

The laminar basement membrane, composed of laminin, collagen IV, nidogen, and perlecan (61–64), is a continuous layer beneath the valve endothelium (9, 63, 64), serving as the interface between endothelial cells and VICs. Laminin, a major basement membrane glycoprotein (61, 62, 65–70), is produced by VECs (71, 72), and present along the endocardial surface of the developing valve cushions during embryogenesis, and the mature mitral leaflets (62, 65). During MV development in mice, laminin is part of the ECM scaffold supporting EndoMT and leaflet formation (72–76). In the adult mice, it remains a thin sheet lining both atrial and ventricular surfaces (71), which maintains endothelial polarity and barrier function, especially in regions of high shear stress, such as the MV (9, 64, 77–79). Autoantibody binding to these components destabilizes endothelial integrity and drives valve matrix injury (45, 48, 49, 54, 56) (Figure 2).

#### 5.1.3 Extracellular matrix proteins as autoantigenic targets

GlcNAc, the group A carbohydrate antigen, shares epitopes with glycoproteins present in valve tissue (57). GlcNAc-reactive antibodies target proteoglycan-rich areas, including the spongiosa layer, which contains GAGs and proteoglycans. This contributes to spongiosa disruption, leaflet swelling, ECM disorganization, and fibrosis (Figure 2). In addition, certain rheumatogenic Streptococcus pyogenes serotypes (e.g., M3 and M18) interact directly with structural collagens in the valve matrix, forming immune complexes that intensify autoimmune injury (Figure 2). Patients with ARF and RMS exhibit significantly elevated antibody titers against both collagen I and collagen IV (59, 60). Anti-collagen I antibodies often accompany anti-myosin responses. These may arise from streptococcal-induced collagen aggregation or the release of collagen from damaged valves during ongoing inflammation. Collagen IV, a key basement membrane component, is also targeted (Figure 2). Furthermore, certain M protein serotypes form immune complexes with collagen IV, promoting endothelial inflammation and matrix breakdown (58). Anti-collagen IV antibodies appear to arise as part of an intrinsic autoreactive process rather than direct molecular mimicry.

#### 5.1.4 Immunopathogenesis of rheumatic mitral stenosis

Based on the prevailing concept of molecular mimicry, GAS induces an aberrant autoimmune response in genetically susceptible hosts. As a result, both humoral and cellular immune pathways are activated against MV tissues, producing the chronic inflammation and fibrosis characteristic of RMS. It is thought that, following GAS infection, B cells mount a vigorous antibody response against streptococcal antigens. Due to antigenic mimicry, antibodies cross-react with host proteins such as cardiac myosin, laminin, collagen, and GlcNAc-containing glycoproteins (42, 45, 48, 49, 54, 56, 80). Many of these autoantigens are expressed on or near the valve endothelium, making the MV particularly vulnerable. Autoantibody binding to the valve surface induces endothelial activation (42, 81, 82), activated endothelium becomes adhesive and permeable, facilitating infiltration of other antibodies, inflammatory cells, and T lymphocytes (83). The formation of immune complexes further promotes neovascularization and lymphangiogenesis within valve leaflets in moderate and severe RMS (84, 85) (Figure 2).

Streptococcal antigens are presented by human leukocyte antigen (*HLA*) class II molecules to T cells, activating autoreactive CD4^+^ (Th1 and Th17) and CD8^+^ T cells (86, 87). Activated T cells secrete pro-inflammatory cytokines, including IL-2, TNF-α, IFN-γ, IL-6, and IL-8, which amplify local inflammation, enhance endothelial activation, and promote ECM remodeling. Th1 cells mediate cytotoxic and pro-inflammatory injury, whereas Th17 cells sustain chronic inflammation and recruit additional immune cells into the affected valve. Elevated IL-6 and IL-8 further stimulate B-cell activation and autoantibody production, while paradoxical upregulation of IL-10 in some patients may promote B-cell survival and differentiation (87), perpetuating the autoimmune response. The post-streptococcal immune cascade in RMS has been extensively characterized; detailed mechanistic descriptions can be found in prior comprehensive reviews (42, 45, 88).

#### 5.1.5 Progression to chronic valvulitis

Previous studies have demonstrated that the initial post-GAS injury manifests shortly after infection, predominantly as MR in children and young adults (48, 56, 88–92). This early regurgitation arises primarily from functional alterations of the MV, including annular dilation, which reduces effective leaflet coaptation and leads to valvular incompetence. Loss of the normally broad coaptation surface increases mechanical tension on the primary CT. As these primary cords are structurally unsuited to sustain excessive tensile stress, they progressively elongate, resulting in impaired apposition between the anterior and posterior mitral leaflets and subsequent prolapse of the anterior leaflet edge into the left atrium (89). In contrast, repeated inflammatory insults resulting from recurrent GAS infections drive the transition from acute injury to chronic inflammation. The combined actions of cross-reactive antibodies, infiltrating T cells, and pro-inflammatory cytokines create a self-perpetuating cycle of endothelial injury and immune activation (93). This vicious cycle leads to progressive ECM deposition, leaflet fibrosis and thickening, chordal shortening and fusion, neovascularization and lymphangiogenesis, and ultimately, calcification and fixed stenosis (Figure 2). Over time, scar formation and structural remodeling result in irreversible valve deformity and the classic morphological features of RMS (57). Therefore, RMS typically develops several years after the initial episode of ARF, and in some cases, may take decades to become clinically apparent (92). This delayed progression aligns with epidemiological observations showing that rheumatic MR predominates in younger patients, whereas RMS becomes increasingly prevalent with advancing age (94).

#### 5.1.6 Genetic susceptibility to rheumatic mitral stenosis

It is estimated that only 3%–6% of individuals with symptomatic GAS infection develop ARF (95), and among those with ARF, approximately 60% progress to RHVD (57). These observations suggest that, beyond the initial GAS infection, host genetic and epigenetic factors play critical roles in determining individual susceptibility to RMS.

Recent genome-wide association studies (GWAS) have identified several loci associated with increased RHVD susceptibility across diverse populations. In a cohort of 2,852 individuals recruited from eight Oceanian countries, a variant within the immunoglobulin heavy chain (*IGH*) locus, specifically the *IGHV4-61*02* allele, is associated with a 1.4-fold increased risk of RHVD (96). A separate GWAS of 1,163 South Asian individuals (672 cases vs. 491 controls) identified the class III region of the *HLA* complex as a major determinant of RHVD susceptibility. The top variant in this region, rs201026476, showed a combined odds ratio of 1.81, and the association was further replicated in European cohorts (97). In Aboriginal Australians (398 RHVD cases and 865 controls), the strongest genetic association was observed at *HLA-DQA1* (rs9272622). Two *HLA* class II haplotypes, *HLA-DQA1*0101_*DQB1*0503* and *HLA-DQA1*0103_*DQB1*0601*, were identified as risk haplotypes, whereas *HLA-DQA1*0301_*DQB1*0402* appeared protective. These findings further support molecular mimicry as a key mechanism in RHVD pathogenesis (98). Furthermore, the RHVDGen study, which enrolled 2,548 RHVD cases and 2,261 ethnically matched controls from eight continental African countries, identified a novel susceptibility locus at 11q24.1 in this first large-scale evidence of a genetic contributor to RHVD in African populations (99).

A complex and population-specific genetic architecture underlies RHVD, highlighting the contributions of both immune-related loci (such as *HLA* and *IGHV*) and novel genomic regions (like 11q24.1) to disease susceptibility. However, some associations identified in specific populations have not been replicated in others. The *HLA* variant rs9272622, reported as the strongest association in Aboriginal Australians, was not tested in the RHVDGen study, while the *HLA* class III variant rs201026476, identified in South Asian and European cohorts, was not genotyped in RHVDGen (99). These discrepancies highlight the influence of geographic, ethnic, and polygenic factors in RHVD pathogenesis, highlighting its complex and heterogeneous genetic mechanisms.

#### 5.1.7 Epigenetic regulation in rheumatic mitral stenosis

Despite extensive GWAS, no single genetic variant has been conclusively identified as a major causal determinant of RHVD. This observation suggests that, beyond inherited genetic susceptibility, epigenetic mechanisms may play a pivotal role in disease initiation and progression, although studies in this field remain limited. Indeed, complementary evidence from genome-wide DNA methylation profiling of peripheral blood samples from six RHVD patients with pulmonary hypertension and six healthy controls supports a role of methylation imbalance in disease pathogenesis (100). Differentially methylated regions (DMRs) were enriched in several genes, including *PRKCA* (protein kinase C α), *PRKAG2* (protein kinase AMP-activated noncatalytic subunit γ2), *SPRED2* (sprouty-related EVH1 domain-containing 2), and *LIF* (leukemia inhibitory factor). Subsequent *in vivo* analysis of RMS valve leaflets revealed hypomethylated CpG islands at the promoter and 5′ terminal regions of *PRKCA*, accompanied by markedly increased *PRKCA* expression and downregulation of its antisense long non-coding RNA (*PRKCA-AS1*) (101). Consistently, chromatin immunoprecipitation (ChIP)-qPCR assays demonstrated that DNA methyltransferase 1 (DNMT1) directly regulates this methylation process, highlighting a lncRNA–DNMT1–PRKCA regulator*y* axis that governs gene transcription in valvular tissue.

Similarly, right atrial (RA) myocardium samples from 73 patients with RHVD and 4 healthy controls showed significant global DNA hypermethylation, as measured by an ELISA-based assay (102). The extent of global methylation positively correlated with patient age, with the strongest association observed in older individuals. Moreover, *ICAM1* mRNA expression was markedly elevated in RHVD patients, coinciding with promoter hypomethylation of the *ICAM1* gene. However, because these analyses were performed in RA myocardium rather than the MV itself, the direct relevance of these findings to rheumatic MV pathology is limited. Given that RHVD predominantly affects the MV, while the RA is comparatively less involved, valve-specific epigenetic profiling will be essential to fully elucidate disease-relevant methylation changes.

*In vitro* studies have shown that antibodies isolated from the sera of RMS patients can directly modulate endothelial epigenetic status. Specifically, RMS patient-derived autoantibodies induce DNA hypomethylation in genes encoding ECM components, including vimentin, laminin B, and ICAM1, as well as in pro-inflammatory cytokines such as IL6 and IL8 within VECs (55). This autoantibody-induced hypomethylation correlates with increased mRNA expression of these genes, suggesting that autoimmune triggers can promote transcriptional activation of ECM and inflammatory pathways through epigenetic dysregulation. Although these findings provide evidence that RMS autoantibodies can alter endothelial methylation patterns, the upstream mechanisms remain unknown. In particular, how RMS autoantibodies influence the function or expression of key DNA methyltransferases (DNMT1, DNMT3A, DNMT3B) or the demethylating enzymes TET1, TET2, and TET3, thereby reshaping the epigenetic landscape of ECM and inflammatory genes, has not yet been defined.

Insights from other autoimmune and inflammatory diseases suggest that similar pathways may be operative in RMS. Global DNA hypomethylation has been documented in peripheral T cells, monocytes, and B cells of patients with rheumatoid arthritis (RA), accompanied by reduced expression of DNMT1 and modest reductions in DNMT3A in B cells (103). Conversely, demethylation enzymes, TET1, TET2, and TET3, are significantly upregulated in monocytes, and TET2 is elevated in T cells. Synovial tissue from RA patients similarly exhibits increased TET3 and 5-hydroxymethylcytosine (5hmC) levels, and cultured fibroblast-like synoviocytes show parallel upregulation (104). In osteoarthritis (OA), chondrocytes display a 5 to 6-fold increase in global 5hmC with concomitant loss of *TET1* (105). Functionally, genetic loss of *Tet1* in mice prevents the development of OA and attenuates disease progression, indicating that TET-mediated demethylation can be a critical driver of chronic joint inflammation and ECM remodeling (106, 107).

These mechanistic analogies related antoimmune and inflammatory disease raise an intriguing hypothesis: whether autoantibody-mediated activation of TET enzymes contributes to pathological demethylation in RMS, and if so, how targeted modulation of TET activity may alter the course of valve injury? For example, loss of *Tet1* may reduce aberrant demethylation, dampen pro-inflammatory signaling, limit ECM remodeling, and potentially facilitate recovery from acute carditis and early valvulitis. Thus, exploring how TET–DNMT balance is perturbed by RMS autoantibodies and whether manipulating this axis can prevent progression from rheumatic inflammation to chronic valvular deformity represents a compelling future research direction. Such studies may ultimately identify new early-intervention strategies to prevent irreversible rheumatic valve remodeling and reduce the long-term need for surgical repair.

#### 5.1.8 Mouse models of rheumatic mitral stenosis

Because GAS infection and molecular mimicry form the pathogenic basis of RMS, efforts to establish mouse models have largely focused on reproducing the ensuing carditis and valvulitis through exposure to GAS or its components, particularly the M protein. As early as 1969, mice injected intraperitoneally with isolated GAS cell wall fragments were reported to develop rheumatic-like cardiac lesions and valvulitis, characterized by diffuse inflammatory infiltrates and nodular lesions within the MV (108). Subsequent studies across different mouse strains—including BALB/c, Swiss, A/J, and DBA/2, using GAS antigens and/or cardiac myosin primarily demonstrated myocarditis with infiltration of CD4^+^ lymphocytes, but notably, no significant valvulitis was observed in these models (109–113).

Given these limitations, more recent work has shifted toward the Lewis rat, which appears more susceptible to developing RHVD-like pathology. Immunization of Lewis rats with recombinant type 5 or type 6 streptococcal M protein rM5 (114–117), rM6 (118) or with formalin-killed GAS (119, 120) induces both myocarditis and valvulitis. In one study, approximately 75% of immunized rats developed rheumatic-like myocarditis, and 62.5% exhibited chronic valvulitis after 24 weeks (120). Histopathological findings include cellular infiltrates, Aschoff-like cells, verrucous vegetations, and chronic lesions such as fibrosis and vascular neogenesis (120). The rM6 injection shows that the valvular lesions were marked by infiltration of CD3^+^, CD4^+^, and CD68^+^ cells, which is consistent with the immune cell composition observed in human RHVD (118). Lesion formation was shown to begin at the valve surface endothelium and progressively extend into the valve matrix (118).

Although some studies have evaluated functional cardiac impairment in these models (116), echocardiographic evidence of valve dysfunction, the hallmark of human RHVD, remains lacking. Since the principal clinical manifestation of RHVD is mitral and/or aortic valve stenosis, with or without regurgitation, the absence of demonstrable valvular dysfunction underscores a major gap between current animal models and human disease pathology. Future animal models should aim to reproduce the progressive valvular remodeling and stenosis characteristic of RHVD. Nevertheless, the currently available rat models utilizing rM5 or rM6 provide a valuable experimental platform to mimic key aspects of M protein–dependent immune activation and offer important opportunities to investigate the early pathogenic mechanisms underlying RHVD.

### 5.2 Congenital mitral stenosis

CMS was first described by Ferencz and colleagues in 1954 (121). It is a rare and morphologically heterogeneous developmental lesion involving both the MV leaflets and the subvalvular apparatus. The reported incidence is approximately 0.6% among autopsied congenital heart defects and 0.21% to 0.42% in clinical series (33). Van Praagh classified CMS into four anatomic subtypes: Type I, typical congenital MS (49%); Type II, hypoplastic MS (41%); Type III, supramitral ring (12%); and Type IV, parachute MV (8%) (33).

Typical CMS is the most common subtype (33). Morphological changes primarily involve thickened, rolled leaflet margins, shortened and/or thickened CTs, partial or complete obliteration of interchordal spaces by fibrous tissue, and underdeveloped PMs with a mildly reduced interpapillary distance (33, 122). Severe cases may present as an arcade MV, in which the PMs fuse and insert directly into the anterior leaflet, forming a muscular arcade. Other cases resemble a hammock MV, where short, thickened chordae insert directly into the posterior ventricular wall, giving the appearance of a hammock when viewed from the LA. Recent reports describe CMS in which both PMs attach directly to the leaflets with minimal or absent chordae (123). Focal endocardial sclerosis of the LV was also observed in this subtype. Despite the obstructive morphology, left ventricular size is typically normal or only mildly reduced (>70% of normal).

Hypoplastic CMS is the second most common subtype of CMS and is generally regarded as part of the hypoplastic left heart syndrome (HLHS) spectrum, as mitral hypoplasia is almost always accompanied by severe left ventricular underdevelopment and aortic outflow obstruction (33, 122). As a result, it is seldom the primary clinical diagnosis. Morphologically, the MV is proportionally small, with a markedly reduced orifice, miniature leaflets, shortened but usually non-thickened CTs, and small PMs that remain normally separated. All valve components appear as scaled-down versions of the normal mitral apparatus.

A supramitral ring is a circumferential shelf of connective tissue arising from the atrial surface of the MV leaflets, distal to the entrance of the LA appendage, and producing varying degrees of obstruction to mitral inflow, with or without other types of CMS (33, 122). This lesion is often accompanied by additional levels of left-sided obstruction, as in Shone's complex, although mild forms have occasionally been reported as *de novo* diagnoses in adults.

The parachute MV is the least common form of CMS and is most often identified in adults (33, 122). In this subtype, the CTs are thickened and shortened and converge onto a single PM—typically the posteromedial—while the anterolateral PM is absent or hypoplastic.

#### 5.2.1 Congenital mitral stenosis cohort studies

Serial reports highlight the rarity and anatomic heterogeneity of CMS. In a 28-year series from 1986 to 2014, 137 children (mean age 4.1 ± 5.0 years; range 1 month to 16.8 years) were diagnosed with CMS, including 56 with typical congenital MS (Type I), 15 with hypoplastic MS (Type II), 48 with a supravalvar mitral ring (Type III), and 10 with a parachute MV (Type IV) (124). A separate study of 108 patients treated between 1985 and 2003 reported a similar distribution: typical CMS in 78 patients, supramitral ring in 46, and parachute MV in 28, with frequent overlap of anatomic lesions (125). Among both cohorts, typical CMS was the most common subtype, followed by supramitral ring, parachute MV, and hypoplastic MS. Therapeutic strategies included balloon mitral valvuloplasty, surgical mitral valvuloplasty, supramitral ring resection, PM splitting or fenestration, and MV replacement, applied according to the underlying morphology (124, 125).

#### 5.2.2 Disease mechanisms in congenital mitral stenosis

Mechanistic investigation of CMS is limited. In a published mouse study, a typical CMS arising from epigenetic dysregulation is the endocardial-specific deletion of *Dicer1* (126), which impairs microRNA processing. *Dicer1*-deficient mice develop hyperplastic MVs with both stenosis and regurgitation, while other valves, such as the tricuspid valve, show only minimal abnormalities. Mechanistically, the loss of *Dicer1* disrupts mesenchymal condensation and ECM remodeling, with dysregulation of more than 20 ECM-related genes, including collagens, proteoglycan core proteins, elastin components, Fibronectin, and Laminins. Single-cell RNA sequencing further revealed the accumulation of immature mesenchymal cell populations in the MV. It highlights the essential role of miRNA-mediated gene regulation in MV development and the pathogenesis of CMS.

The second major subtype is hypoplastic MS, which often co-occurs with HLHS and is rarely observed as an isolated defect. Consequently, most genetic studies focus on HLHS cohorts. Recent evidence links KMT2D, a chromatin-modifying histone methyltransferase (H3K4 methylation), to congenital MV disease. In a reported case, an infant with severe HLHS—including mitral and aortic atresia, carries a somatic mosaic *de novo* nonsense variant in KMT2D (c.8200C>T, p.R2734*) (127). This mutation is associated with an aberrant methylation pattern characteristic of Kabuki syndrome, suggesting that disturbance of chromatin modification pathways may drive severe MV hypoplasia and stenosis. Beyond individual case reports, larger sequencing studies reinforce this connection. Among 48 HLHS patients, 33 exhibited either MS or mitral atresia (MA), and six of them harbored pathogenic variants: four in KMT2D, one in NOTCH1, and one isolated mitral atresia case with a KMT2D variant (128). These findings support the hypothesis that chromatin-mediated epigenetic dysregulation contributes to MV hypoplasia.

KMT2D modifies chromatin by depositing H3K4 methylation, a mark associated with active enhancers and promoters. Loss of KMT2D disrupts transcriptional access across many tissues. Multiple conditional mouse models have been generated to investigate the role of *Kmt2d* in cardiovascular development. Early cardiac progenitor deletion using *Mesp1*-Cre (129) or *Mef2c*-anterior heart field (AHF)-Cre (129) produces embryonic lethality with severe hypoplastic hearts, outflow tract abnormalities, and disrupted interventricular septation. Deletion in cardiomyocytes using *Tnnt2*-Cre (129) results in a thin compact myocardium, impaired cardiomyocyte proliferation, and developmental delay, demonstrating that myocardial *Kmt2d* is required for early cardiac growth. Beyond embryogenesis, inducible cardiomyocyte-specific loss of *Kmt2d* in adults leads to worsened cardiac function and increased vulnerability to myocardial ischemic injury (130, 131), indicating a continuing role for KMT2D in cardiac homeostasis. Although these models establish that KMT2D is essential for both early cardiac morphogenesis and adult myocardial integrity, there are currently no published reports of endocardial-specific *Kmt2d* deletion (e.g., *Nfatc1*-Cre, *Tie2*-Cre) to directly examine its function in endocardial-derived valve mesenchyme. This gap highlights that the contribution of KMT2D to MV development and congenital mitral hypoplasia remains largely unexplored.

## 6 Myxomatous mitral valve prolapse

MMVP, originally characterized by Barlow in the early 1960s (132, 133), is now recognized as the leading cause of severe non-ischemic mitral regurgitation (MR) in the United States (134). Clinically, MMVP is defined echocardiographically by the systolic billowing of one or both mitral leaflets into the LA, extending beyond the mitral annular plane on the parasternal long-axis view (135). Although the overall clinical outlook for individuals with MMVP is generally favorable, a minority of patients experience important complications, including infective endocarditis, malignant arrhythmias with risk of sudden cardiac death, and progression to severe MR (134). Population-based studies estimate a prevalence of roughly 1.3% to 1.4%, whereas hospital-based cohorts report markedly higher rates, approaching 8% to 9% (136). MMVP can be detected across all age groups—sometimes even in infancy—and its frequency increases with aging, reaching nearly 3% in older adults (136). Most of the affected individuals do not develop hemodynamically significant regurgitation and will never require surgical or catheter-based intervention. Nevertheless, up to one-quarter of patients eventually progress to clinically significant MR (137). The overall incidence of all-cause mortality in MMVP cohorts is estimated at approximately 1.7 events per 100 person-years, underlining that the majority of patients have a benign disease course (136).

### 6.1 Histology change of myxomatous mitral valve prolapse

The defining pathological feature of MMVP is myxomatous degeneration, which produces thickened, redundant leaflets with a soft, gelatinous quality and systolic ballooning into the LA. Both leaflets may be involved, although the posterior leaflet is affected far more frequently (≈52%) than the anterior leaflet (≈12%) (138). These morphological alterations arise from widespread disturbances in collagen architecture across the leaflet layers as well as abnormalities of the CTs. Typical structural changes include interchordal hooding, elongation or rupture of the chordae, and dilation of the mitral annulus (138, 139).

**Figure 3:**
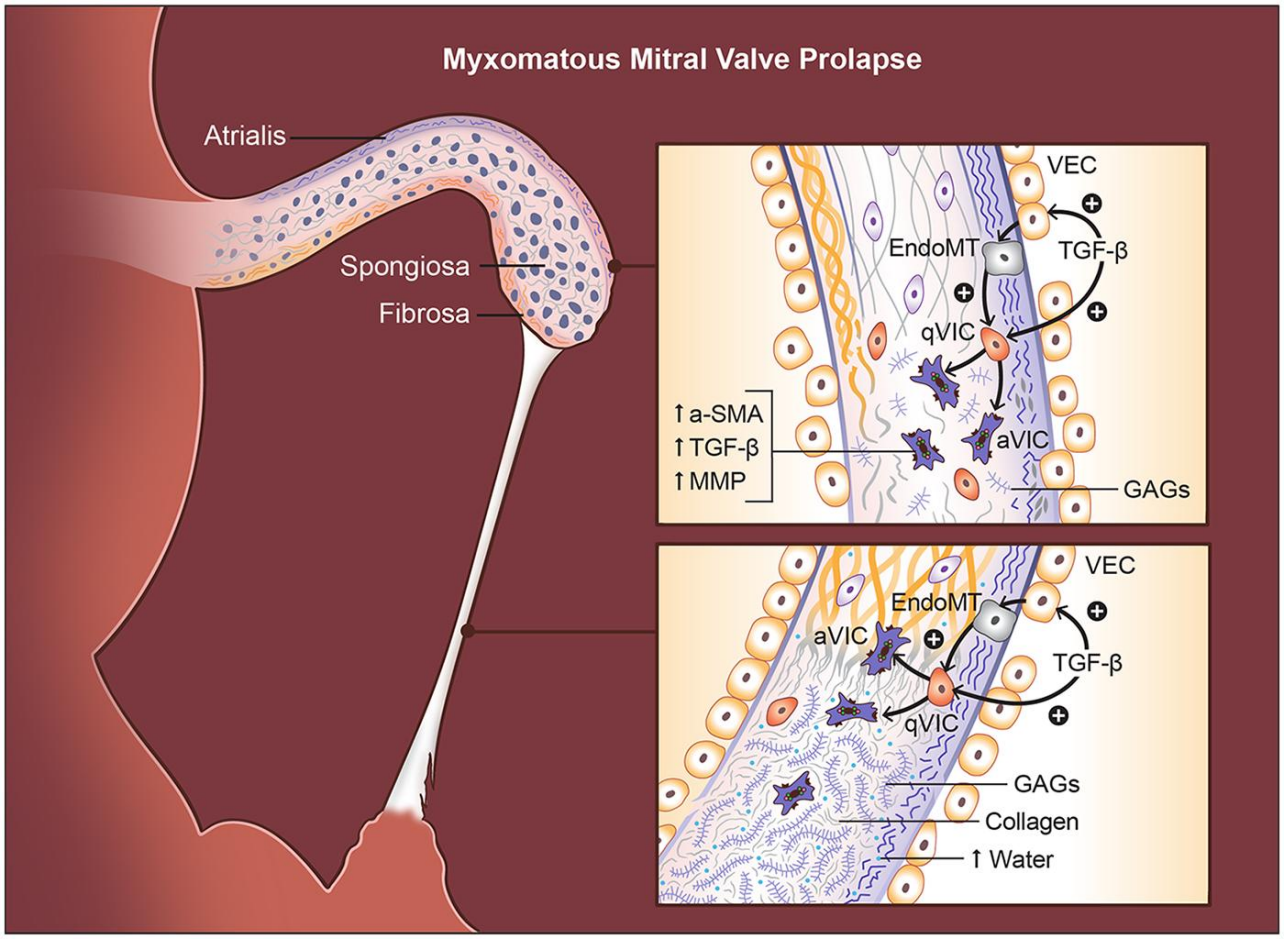
Histopathological and cellular changes in myxomatous mitral valve prolapse (MMVP). Myxomatous degeneration is characterized by multilayered structural disruption. In the atrialis, elastic fibers become fragmented, while in the fibrosa, collagen bundles are disorganized and infiltrated by myxoid matrix. The spongiosa is markedly expanded and filled with loose, proteoglycan-rich extracellular matrix, accompanied by reduced collagen and fragmented elastin. TGFβ signaling drives endothelial-to-mesenchymal transition (EndoMT) in valve endothelial cells (VECs) and activates valve interstitial cells (VICs), increasing BMP and MMP expression, promoting proteoglycan accumulation, and inducing collagen disorganization that leads to leaflet thickening. Chordae tendineae (CT) undergo parallel pathological changes, including diffuse proteoglycan deposition, expansion between collagen bundles, and fragmentation of elastic and collagen fibers. These alterations weaken tensile strength, resulting in chordal elongation or rupture. Compared with normal chordae, myxomatous chordae contain longer glycosaminoglycans, higher water content, and a greater proportional increase in proteoglycans than observed in leaflets.

Histologically, MMVP is defined by marked expansion of the spongiosa, driven by excessive proteoglycan accumulation, and by replacement of more than half of the dense fibrous lamina with myxoid ECM (138) (Figure 3). Within the leaflet, several layers show characteristic alterations. The atrialis often exhibits fibrotic remodeling (140), whereas the fibrosa becomes disrupted and infiltrated by myxomatous matrix, compromising its normal load-bearing collagen architecture (15, 141). The spongiosa is markedly enlarged and filled with loose, amorphous proteoglycan-rich ECM, accompanied by a reduction in collagen content and fragmentation of elastic fibers (140). These structural changes are further exacerbated by increased expression of matrix-degrading enzymes, including MMP1, MMP2, MMP9, MMP13, and cathepsin K, which promote ongoing breakdown of collagen and elastin (140, 142, 143). The elastic lamina beneath both the atrialis and spongiosa layers is frequently lost or severely disrupted, contributing to the overall mechanical vulnerability of the myxomatous leaflet. A hallmark consequence of these ECM alterations is significant leaflet thickening, largely driven by spongiosa expansion and plaque-like accumulation of myxoid tissue.

Myxomatous remodeling in MMVP extends well beyond the valve leaflets themselves. Up to 85% of affected individuals exhibit myxoid infiltration of the mitral annulus, and nearly one-third show involvement of the CTs (138, 139) (Figure 3). Notably, inflammatory cell infiltration is typically absent, highlighting that MMVP represents a degenerative, rather than inflammatory, matrix disorder. A major clinical consequence of this degenerative process is chordal rupture, which occurs in approximately 43.5% of patients with floppy MVs and frequently triggers acute MR (138, 139). The entire chordal system is structurally compromised, with the chordal insertion sites on the leaflets showing the most pronounced degeneration.

Histologically, myxomatous CTs demonstrate striking alterations in connective-tissue architecture (Figure 3). Proteoglycans accumulate throughout the matrix, and the outermost layer displays fragmentation of elastic fibers and collagen fibrils (15). The normally compact spaces between collagen bundles become expanded and filled with abnormal proteoglycans or large proteoglycan aggregates, disrupting the highly organized fibrillar structure required for tensile strength (15). Loss of mature, highly cross-linked collagen and its replacement by immature, poorly cross-linked fibrillar collagen is observed throughout all layers of the CTs, contributing to their reduced tensile strength and increased susceptibility to elongation and rupture (144). Similar deposits surround degenerated elastic and collagen fibers, emphasizing the widespread ECM disorganization that weakens the chordal apparatus.

These qualitative changes are mirrored by more pronounced quantitative alterations in myxomatous CTs than in the leaflets (143) (Figure 3). Myxomatous CTs contain 3% to 7% more water than normal chordae, reflecting their loose, hydrated ECM (143). They also exhibit substantially elevated concentrations of GAGs, and the GAG chains themselves are considerably longer than those found in healthy chordae (143, 145). Notably, the relative increase in GAG content is far greater in the chordae than in the leaflets, emphasizing that CTs experience a more extreme form of myxoid remodeling (143). This excess of water and proteoglycan-rich matrix, together with a relative loss of organized collagen, markedly weakens the tensile strength of the chordae and predisposes them to elongation and rupture (146). Biomechanically, both myxomatous leaflets and chordae show increased extensibility and reduced stiffness compared with normal tissue; however, the decline in stiffness and failure strength is substantially more severe in the chordae. These changes align with clinical observations showing that chordal elongation and rupture represent the most common mechanisms driving severe regurgitation in myxomatous MMVP (147). In this context, the chordae—not the leaflet—often constitute the critical point of structural failure that precipitates clinically significant MR.

As MMVP advances, the myxomatous expansion of the proteoglycan-rich ECM not only disrupts leaflet structure and contributes to regurgitation but also imposes secondary effects on the underlying myocardium. A well-recognized feature of advanced disease is the development of regionalized LV fibrosis, most notably affecting the PMs and the inferobasal LV wall (148). These focal fibrotic changes are believed to result from excessive mechanical stress transmitted to the PMs and adjacent myocardium during systole, exacerbated by abnormal mitral annular dynamics. At the cellular level, myocardial fibrosis is marked by the transformation of resident fibroblasts into α-smooth muscle actin (αSMA)-positive myofibroblasts, which generate contractile stress fibers and produce large quantities of ECM proteins, particularly collagens (149). Over time, these processes lead to fibrosis concentrated in regions mechanically coupled to the prolapsing valve, resulting in a pattern of myocardial remodeling characteristic of MMVP. From an ECM perspective, collagens are the dominant structural elements of cardiac connective tissue. In the normal human myocardium, collagen I and collagen III are the major fibrillar types, comprising approximately 85% and 11% of total collagen content, respectively. In myocardial fibrosis, regardless of etiology, this collagen I/collagen III ratio becomes altered, reflecting changes in collagen synthesis, crosslinking, and degradation (150). In MMVP-associated myocardial remodeling, these shifts further increase the stiffness of ventricular regions subjected to chronic mechanical overload.

It is worth mentioning that mitral annular disjunction (MAD) is frequently observed in patients with MMVP, with reported prevalence ranging from approximately 22% to 90% (151–155). MAD was first described by Bharati and colleagues in 1980 (156) and is defined as a macroscopic separation between the mitral annulus supporting the posterior mitral leaflet and the adjacent left ventricular (LV) myocardium (157). MAD extends laterally beneath the posterior leaflet, involving all posterior scallops but most commonly and prominently affecting the middle scallop or P2 of the posterior leaflet. The severity of MAD correlates with the number of prolapsing mitral leaflet segments in patients with MMVP (158). Accumulating evidence suggests that MAD contributes to adverse LV remodeling, characterized by increased LV dimensions and structural myocardial alterations (159). It may be present with LV myocardial atrophy or replacement fibrosis associated with disease stage and mechanical stress (153, 160). Progressive MAD is also linked to peri-annular inflammation and the development of basal LV fibrosis (161), changes that are increasingly recognized as predisposing to malignant ventricular arrhythmias in patients with MMVP (162).

The structural consequences of myxomatous degeneration provide a unified mechanism for the development of clinically significant MR in MMVP (Figure 3). As the valve leaflets thicken and become increasingly redundant, their exaggerated systolic billowing imposes greater mechanical load on the CTs, which are themselves weakened by proteoglycan accumulation, disorganized collagen, and degeneration of elastic fibers. This imbalance between a heavier leaflet and a structurally fragile chordal apparatus leads to progressive chordal elongation and, in advanced disease, chordal rupture—events that commonly precipitate acute or severe MR. The resulting regurgitant volume further increases mechanical stress on left ventricular regions coupled to the prolapsing valve, contributing to localized myocardial fibrosis, particularly within the PMs and inferobasal LV wall. Thus, although leaflet degeneration initiates the disease, the ultimate severity of MR is largely dictated by the extent of chordal failure, which often determines the outcome of the disease and the need for surgical intervention.

### 6.2 Molecular mechanism of the myxomatous mitral valve prolapse

The structural and functional integrity of the MV is maintained primarily by two resident cell populations: VECs and VICs (146, 163). Under homeostatic conditions, VECs form a protective endothelial monolayer that regulates permeability, inflammation, and paracrine signaling, while simultaneously helping maintain VICs in a quiescent, matrix-preserving state (164). VICs are the predominant cell type within the valve leaflets and populate all three layers, fibrosa, spongiosa, and atrialis, with the spongiosa containing the greatest density of VICs (165), and are viewed as essential for the production of layer-specific ECM (166). qVICs exhibit low contractility, minimal αSMA expression, and limited collagen I production, reflecting their role as custodians of a balanced ECM. In MMVP, however, this equilibrium is disrupted. Two major processes drive the emergence of aVICs (Figure 3): (1) aberrant activation and transdifferentiation of VECs into mesenchymal-like cells through EndoMT, and (2) proliferation and phenotypic activation of resident qVICs (164, 167), both of which give rise to contractile, matrix-remodeling aVICs that are central to degenerative disease progression.

#### 6.2.1 TGFβ-dependent EndoMT as a driver of myxomatous remodeling

At the molecular level, TGFβ is a central regulator of pathological endothelial activation in myxomatous MV degeneration (Figure 3). TGFβ1 stimulation induces EndoMT in VECs, characterized by the loss of endothelial markers and the acquisition of mesenchymal traits, including increased expression of αSMA, Snail, Slug, and MMP2 (168, 169). These changes generate additional mesenchymal-like cells with enhanced contractile and matrix-remodeling capacities (168, 169). Importantly, EndoMT can be suppressed by TGFβ receptor I kinase inhibition, which normalizes αSMA, CD31, and Has2 levels, demonstrating that EndoMT in VECs is directly dependent on TGFβ signaling (170).

Endocrine and paracrine cues further potentiate EndoMT. Osteoprotegerin (OPG), secreted by human MMVP-derived VECs undergoing EndoMT, functions as an autocrine amplifier of endothelial transformation, increasing reactive oxygen species (ROS), accelerating endothelial migration, and stimulating VIC proliferation. These effects expand the pool of aVICs capable of producing proteoglycans, MMPs, and Cathepsins, and elevated plasma OPG levels in MMVP patients position it as both a functional mediator and a circulating biomarker of early disease. Aldosterone similarly drives EndoMT and proteoglycan secretion in VECs, whereas mineralocorticoid receptor blockade abolishes these responses (171), highlighting a broader endocrine axis that interfaces with TGFβ–dependent endothelial plasticity.

Transcriptional profiling from a canine MMVP model provides further support for the involvement of EndoMT (172). Myxomatous valves exhibit significant upregulation of EndoMT-associated genes, including *ACTA2, SNAI1, CTNNB1, and HAS2* (1.38- to 10.66-fold increases), alongside reduced expression of the endothelial adhesion molecule *CDH5* (−2.59-fold), a shift that favors endothelial detachment, migration, and mesenchymal conversion. Together, these findings position TGFβ–dependent EndoMT signaling as a major upstream driver of endothelial plasticity and remodeling in MMVP, providing a molecular link between endothelial activation and the expansion of pathogenic VIC populations.

#### 6.2.2 TGFβ-dependent VIC activation

Extensive *ex vivo* and *in vitro* studies demonstrate that TGFβ signaling is the principal upstream driver of VIC activation in MMVP (Figure 3). In a canine *ex vivo* MMVP model, stimulation of VICs with TGFβ isoforms induces marked upregulation of αSMA, SM22α, versican, and TGFβ ligands themselves, accompanied by substantial proteoglycan accumulation, with TGFβ1 exerting the strongest activation (170). Additional canine studies show that TGFβ3 likewise increases αSMA expression and proteoglycan content in VICs, promoting their transition from qVICs into aVICs, effectively recapitulating hallmark features of myxomatous degeneration. Human VIC culture systems mirror these findings. In MMVP-derived VICs, TGFβ2 suppresses ADAMTS-1 and drives versican accumulation, consistent with ECM abnormalities observed in human myxomatous leaflets (173). Knockdown of metallothionein1/2 (MT1/2) further enhances TGFβ2 expression, reinforcing the pathway's autoregulatory behavior (173). TGFβ2 also increases β-catenin at the mRNA and protein levels, revealing crosstalk between TGFβ and WNT signaling in driving profibrotic and procalcific cellular programs (174). As expected, TGFβ2–treated VICs show strong activation of canonical SMAD2 signaling and induction of multiple profibrotic genes. Across models, these pathological responses are abolished or markedly reduced by TGFβ receptor I kinase inhibition or a pan-TGFβ neutralizing antibody, both of which suppress αSMA expression in VICs derived from myxomatous valves (170). These results place TGFβ signaling at the center of the VIC activation cascade. The therapeutic importance of these pathways is underscored by pharmacologic studies. In human VICs, TGFβ-induced ECM synthesis requires SMAD2/3 and p38 MAPK signaling (175), and this profibrotic response is blocked by angiotensin II receptor blockers (ARBs), demonstrating that renin–angiotensin signaling amplifies TGFβ activity. Similarly, in canine VICs, ACE inhibitors attenuate TGFβ3-driven VIC activation and reduce upregulation of TGFβ3 itself (176), suggesting renin–angiotensin blockade may interrupt both ligand production and TGFβ–dependent VIC activation. Complementary mouse VIC studies demonstrate that cell-permeable antioxidants attenuate TGFβ1-induced profibrotic and matrix-remodeling gene expression, positioning oxidative stress as a cooperating stimulus that amplifies TGFβ-dependent VIC activation (177).

The mouse model of Marfan syndrome (*Fbn1* mutation) provides the clearest demonstration that excess TGFβ activity is both necessary and sufficient to induce myxomatous leaflet thickening (178). Transcriptomic analyses of *Fbn1* mutant valves show robust upregulation of TGFβ-responsive genes, including βIGH3, endothelin-1, and TIMP-1, which are associated with fibrosis, ECM turnover, and VIC activation (178). Notably, BMP-2, BMP-4, BMP-6, and TGFβ2 are also elevated, consistent with TGFβ-driven positive-feedback amplification through both autoinduction and cross-regulation of BMP pathways. TGFβ antagonism significantly reduces leaflet thickness and length in all genotypes, revealing a direct causal relationship between heightened TGFβ signaling and myxomatous degeneration. The result is a sustained TGFβ/BMP-rich environment that promotes proteoglycan accumulation, collagen disorganization, and VIC activation, producing the characteristic thickened, myxomatous leaflet (178).

Additional mouse models highlight the importance of TGFβ hyperactivation. *Ltbp2*-deficient mice, which lack a key extracellular TGFβ regulator, exhibit overexpression of downstream TGFβ targets, including periostin, runx2, and CTGF, demonstrating valve-intrinsic TGFβ pathway enhancement (179). Similarly, conditional deletion of *Fstl1* in endothelial/endocardial lineages results in sustained TGFβ and BMP signaling, excessive proliferation, culminating in deformed MVs, MR, diastolic dysfunction, and early lethality (180). These models illustrate how loss of endogenous inhibitors of TGFβ/BMP signaling leads to progressive valve malformation and functional failure. Late-stage MMVD also shows increased phospho-Smad2/3, the canonical TGFβ signaling readout, primarily within the atrialis layer, together with elevated Bax and reduced Bcl-2, indicating TGFβ–linked apoptosis and structural compromise (181), and TGFβ1 emerges as the top upstream regulator, alongside TNF and IFN-γ, regardless of disease stage (182). Canine MMVD, a translationally relevant spontaneous model, expresses significant grade-dependent increases in hallmark disease genes such as *Acta2, Htr2b, Mmp12, and Cdkn2a*, with functional enrichment dominated by ECM remodeling and inflammation. Of note, the expression of TGFβ3, TβR-II, αSMA, and MMP3 is detectable only in diseased canine valves (176).

*Axin2*-deficient mouse models further reveal interactions between Wnt/β-catenin signaling and TGFβ/BMP pathways. *Axin2*-deficient mice, which exhibit heightened β-catenin activity, develop enlarged mitral and aortic valves with reduced collagen deposition, increased proliferation, elevated BMP signaling, and progressive myxomatous degeneration resembling human disease (183). Consistent with these findings, human MMVP-associated variants, lead to increased nuclear β-catenin, enhanced transcription of targets such as MMP2, collagen matrix loss, and a myxomatous phenotype (184). These studies highlight the convergence of β-catenin signaling with TGFβ/BMP–mediated ECM remodeling in MMVP pathogenesis.

Collectively, the animal studies demonstrate that TGFβ hyperactivation is a unifying and indispensable driver of myxomatous MV degeneration. Across diverse models-whether triggered by genetic mutations (*Fbn1*, *Ltbp2*, and *Fstl1*), dysregulated β-catenin signaling, or spontaneous degenerative disease in canine MMVD-heightened TGFβ activity consistently leads to VIC activation, proteoglycan-rich matrix expansion, collagen fragmentation, ECM disorganization, and the progressive thickening and deformity of valve leaflets. These *in vivo* observations mirror the pathological processes identified in *ex vivo* experiments and confirm that the cellular consequences of TGFβ signaling translate directly into whole-organ structural remodeling. Together, this body of evidence firmly establishes TGFβ signaling as a central pathogenic mechanism and a high-priority therapeutic target in the prevention and treatment of MMVP.

#### 6.2.3 Human myxomatous valves

Human surgical specimens from myxomatous MVs offer definitive evidence that the molecular pathways identified *in vitro* and in animal models are fully operative in clinical disease. aVICs appear earliest as clusters in the subendocardial atrialis and progressively expand into deeper layers of the leaflet as disease severity increases (185). These cells exhibit a classical myofibroblast phenotype, expressing αSMA, vimentin, and nonmuscle myosin heavy chain B, consistent with enhanced contractility and matrix-remodeling capacity (185). At the molecular level, TGFβ hyperactivation is a prominent feature of human MMVP. Enhanced TGFβ1, *p*-Smad2, TIMP1, CTGF, and Cadherin-11 expression was found in human myxomatous leaflets (186). TGFβ2 expression is significantly elevated in human myxomatous valves, accompanied by a striking downregulation of MT1/2 and ADAMTS proteases, both of which normally contribute to redox homeostasis and ECM turnover (173). Myxomatous valves also demonstrate activation of canonical TGFβ signaling, reflected by increased SMAD2/3 phosphorylation (175, 177), as well as upregulation of BMP4-mediated pathways and Wnt/β-catenin signaling (187), the latter marked by increased expression of their common profibrotic target Runx2 (174). These convergent pathways highlight a broad, integrated disturbance of developmental and fibrotic signaling in the diseased MV. Human myxomatous leaflets further exhibit strong expression of matrix-modifying enzymes, including collagenases (MMP1 to MMP13), gelatinases (MMP2, MMP9), and cysteine proteases (cathepsins C and M) (140, 142, 185). Pro-inflammatory mediators such as IL-1β amplify these degradative processes (140, 188). Although aVICs maintain the ability to synthesize collagen, demonstrated by robust procollagen-I mRNA expression (140), the rate of collagen fragmentation and elastin breakdown far exceeds synthesis, leading to progressive weakening of the fibrosa and disorganization of the leaflet core.

#### 6.2.4 Mechanical stress-dependent EndoMT and VIC activation

Mechanical stress acts as a potent biological stimulus in MMVP and appears to reactivate embryonic valve developmental programs. Experimental and clinical evidence demonstrates that stretched or tethered leaflets show a dramatic increase in CD31^+^ endothelial cells coexpressing αSMA, compared with quiescent, unstressed valves (41 ± 19% vs. 9 ± 5%) (167). This finding indicates robust mechanically induced EndoMT. In stressed valves, αSMA^+^ cells accumulate along the atrial endothelial surface and penetrate the valve interstitium, where they correspond with regions of increased collagen deposition, suggesting active fibrosis. Similar remodeling occurs within the CTs: stretched CTs exhibit endothelial αSMA expression, subendothelial clusters of αSMA^+^ myofibroblasts, and loss of normal collagen alignment and density (167). These cellular responses highlight the valve's inherent mechanoresponsiveness, mechanical overload not only initiates EndoMT but also expands the population of aVICs, transforming the MV into an actively remodeling and mechanically adaptive tissue, consistent with the progressive changes seen in MMVP.

#### 6.2.5 Genetics of myxomatous mitral valve prolapse

The genetic basis of MMVP is only partially understood, but both syndromic and nonsyndromic forms have been described. Syndromic MMVP occurs in the context of several heritable connective-tissue disorders, most notably Marfan syndrome (caused by mutations in *FBN1*) (189) and Ehlers-Danlos syndromes, which involve pathogenic variants in collagen genes (*COL1, COL3, COL5, COL11*) and *TENASCIN* (190). Additional associations include pseudoxanthoma elasticum (PXE), resulting from mutations in *ABCC6* (191), Loeys-Dietz syndrome caused by mutations in *TGFBR1* or *TGFBR2* (189), *Filamin A* (*FLNA*) mutation syndrome linked to *FLNA* mutations (192), Williams-Beuren syndrome due to *ELASTIN* (*ELN*) deletions (193), aneurysm-osteoarthritis syndrome driven by *SMAD3* mutations (194), and juvenile polyposis syndrome involving *SMAD4* variants (195). The focus of this part is nonsyndromic, degenerative MMVP, which comprises the majority of MMVP cases and arises independently of systemic connective-tissue disorders. Four causal genes (*FLNA, DCHS1, DZIP1, TNS1*) have been identified through multiple sequencing and genomics studies for nonsyndromic MMVP.

*FLNA* was the first gene identified as causal for isolated, nonsyndromic, X-linked MMVP (196). *FLNA* encodes a large cytoskeletal scaffolding protein that crosslinks actin filaments and anchors them to membrane receptors and signaling complexes (197). Through these interactions, FLNA functions as a mechanotransduction hub, converting mechanical forces into biochemical signals and coordinating cytoskeletal remodeling (198). This role is particularly critical during fetal valve morphogenesis, where FLNA expression is enriched in developing leaflet interstitial cells. Genetic studies have identified multiple pathogenic missense mutations in *FLNA* in families with nonsyndromic MMVP. Early linkage and positional cloning analyses uncovered the familial P637Q mutation (196, 199), followed by additional variants including G288R, H743P, and V711D (196, 199, 200), all of which segregate with valvular dystrophy in multiple extended pedigrees. A novel splice-site mutation (c.1066-3C>G) was later identified (201), producing an in-frame deletion of eight amino acids near the N-terminal domain and cosegregating with an isolated, male-expressed MMVP phenotype without skewed X-inactivation in female carriers. Mechanistic studies have revealed how *FLNA* mutations disrupt normal cytoskeletal signaling. In a yeast two-hybrid screen, PTPN12 (PTP-PEST) was identified as a specific binding partner of FLNA (202). MMVP-associated FLNA mutations (G288R, P637Q, H743P) impair FLNA-PTPN12 binding and reduce activation of downstream PTPN12 effectors, including Src and p190RhoGAP, which are key regulators of focal adhesion dynamics and actin organization. These defects weaken the valve's ability to sense and respond to mechanical strain, suggesting that impaired mechanotransduction is a core driver of FLNA-related MMVP. Structural studies of the P637Q mutant further demonstrate that even subtle conformational changes can significantly alter FLNA's capacity to transmit cellular forces and engage signaling partners (203). Mouse models provide definitive functional validation of FLNA's role in maintaining MV integrity. *Flna*-deficient valves exhibit marked ECM disorganization, impaired interstitial cell–mediated matrix remodeling, and structural features characteristic of myxomatous leaflet degeneration (204). Mechanistic studies further identify a serotonin–transglutaminase-2–FLNA axis that operates during fetal valve development, linking FLNA-dependent cytoskeletal regulation to proper ECM organization (204). In *Tie2*-Cre;*Flna* ^flox/flox^ mice, loss of *Flna* in the endocardial lineage results in progressive leaflet thickening, excessive ECM accumulation, and ineffective matrix remodeling, recapitulating the core histopathological hallmarks of MMVP (205). At the signaling level, *Flna* deficiency disrupts the balance between *Erk* and *Smad* pathways and compromises ECM turnover through a β1-integrin–dependent mechanism, accentuating *Flna*'s essential role as a cytoskeletal mechanotransducer that integrates intracellular force transmission with ECM homeostasis (205). More broadly, reduced FLNA expression in human valves correlates with increased ECM hyalinization, particularly in aging male patients, and appears partially mitigated by upregulation of SOX9, a key transcription factor governing ECM integrity (206).

*DCHS1* encodes a large cadherin-repeat–containing cell-adhesion molecule essential for planar cell polarity (PCP), tissue morphogenesis, and coordination of mechanical forces during cardiac development. During fetal and neonatal stages, DCHS1 exhibits dynamic subcellular localization, marking non-myocyte, non-endothelial cells that extend polarized projections bridging endothelial and interstitial cell populations—behaviors consistent with its proposed role as a molecular tether mediating heterotypic and homotypic interactions within the developing leaflet architecture (207). Linkage analysis of three multigenerational families with autosomal-dominant MMVP identified two pathogenic variants, R2513H and R2330C, both of which demonstrate loss-of-function in cell-based assays and impair DCHS1-mediated mechanical cohesion (208). Functional modeling across species confirms the evolutionary conservation of DCHS1 function. In zebrafish, morpholino knockdown of *dchs1* causes atrioventricular regurgitation, failure of atrioventricular constriction, and abnormal endocardial cushion formation. In *Dchs1*^+^^/^^−^ mice, partial deficiency produces hallmark myxomatous features, including leaflet prolapse, thickened and disorganized leaflets, and excess proteoglycan accumulation, reflecting impaired fibrosa integrity and aberrant ECM patterning (208). Mechanistically, DCHS1 operates within a broader structural module. A DCHS1-LIX1L-SEPT9 (DLS) protein complex was identified through yeast two-hybrid screening, revealing its role in promoting filamentous actin assembly, cell–ECM alignment, and proper leaflet morphogenesis. Compound-heterozygous mice (*Dchs*1^+^^/^^−^; *Lix1l*^+^^/^^−^) exhibit marked leaflet enlargement at birth and in adulthood, reinforcing the functional interdependence of this pathway in shaping valve geometry (209). Human genetic studies continue to expand the mutational spectrum of *DCHS1*. Sequencing of 100 unrelated MMVP cases identified 24 carriers of predicted deleterious missense variants, including a novel (A2464P) and two rare variants (R2770Q and R2462Q) (210). Additionally, whole-exome sequencing (WES) has uncovered a *de novo* splice-site mutation (c.6364+1G>C) associated with MMVP (211). So DCHS1 is a developmental and structural regulator whose disruption leads to impaired cellular orientation, defective cytoskeletal organization, and ultimately, the myxomatous remodeling and leaflet prolapse characteristic of nonsyndromic MMVP. *DZIP1* encodes a zinc-finger protein localized to the basal body of primary cilia and the nucleus, where it modulates several developmental pathways, including Hedgehog and Wnt/β-catenin signaling (212–214). Its role in nonsyndromic MMVP was first established through WES of four multigenerational families with MMVP, which identified a single heterozygous missense mutation resulting in a serine-to-arginine substitution in known *DZIP1* isoforms (S70R/S24R), strongly supporting *DZIP1* as a causal gene (215). Functional modeling supports this conclusion: *Dzip1* ^S14R/+^ knock-in mice develop classic myxomatous MV degeneration and functional prolapse. Valve-specific deletion of *Dzip1* using *Nfatc1*-Cre further demonstrated that loss of *Dzip1* in valve mesenchyme progenitor cells results in shortened primary cilia, impaired mechanosensation during development, and myxomatous leaflet thickening characterized by increased proteoglycans, collagen disorganization, and loss of normal ECM. Proteomic analyses identified Chibby-1 (CBY1) as a key DZIP1-binding partner, revealing a multimeric DZIP1-CBY1-β-catenin complex that is essential for normal valve morphogenesis (184). This complex localizes to the basal body of primary cilia and functions as a regulatory checkpoint that restrains β-catenin activity during cardiac development. Disruption of the complex, either through inherited mutations or experimental perturbation, destabilizes both DZIP1 and CBY1, leading to unchecked β-catenin signaling. In *Dzip* ^S14R/+^ knock-in mice, increased nuclear β-catenin accumulation drives upregulation of canonical downstream targets such as MMP2, promoting excessive ECM remodeling and collagen degradation. Importantly, a pathogenic *DZIP1* missense variant (C585W) identified in a family with autosomal dominant MMVP maps precisely to the DZIP1-CBY1 interaction domain, providing compelling human genetic evidence that disruption of this cilia-associated regulatory complex is an important mechanistic driver of nonsyndromic MMVP.

*TNS1*, which encodes tensin-1, is a focal adhesion protein that links the ECM to the cytoskeleton and participates in a wide range of cellular processes, including cell adhesion, migration, proliferation, mechanical sensing, and cytoskeletal organization (216). Like FLNA (199), TNS1 interacts with actin and functions in structural integrity and mechanotransduction, making it a biologically plausible contributor to MV architecture and resilience. GWAS have repeatedly identified *TNS1* as one of the strongest genetic susceptibility loci for nonsyndromic MMVP. In two large GWAS comprising 1,412 MMVP cases and 2,439 controls, followed by replication in an additional 1,422 cases and 6,779 controls, the lead variant rs12465515 at chromosome 2q35, located in an intergenic region near *TNS1*, shows genome-wide significant association (217). The second meta-analysis, combining three GWAS (a total of 1,920 MMVP cases and 6,858 controls), again identified the strongest signal at the *TNS1* locus, including variants rs12465515 and rs7595393 (218). Functional modeling supports the pathogenic relevance of TNS1. Morpholino knockdown of *tns1* in zebrafish induced atrioventricular valve regurgitation, demonstrating a conserved requirement for *tns1* in valve function (217). Likewise, *Tns1*^−/−^ mice develop hallmark features of myxomatous degeneration, including increased proteoglycan accumulation and loss of normal ECM stratification, validating *Tns1* as a causal contributor to valve remodeling (217).

In addition to the four well-studied causal genes above, a WGS of 284 unrelated MMVP probands identified *ARHGAP24* as a new gene associated with progressive posterior MMVP (PostMMVP) (219). *ARHGAP24* encodes FilGAP, a Rho GTPase–activating protein and filamin A-binding partner involved in cytoskeletal tension and cell–matrix adhesion. Loss-of-function *ARHGAP24* variants identified in familial cases impaired cellular adhesion and mechanotransduction *in vitro*. Consistent with its functional role, silencing of *arhgap24* in zebrafish produced atrioventricular regurgitation, providing *in vivo* support for pathogenicity. In addition, one WES of 80 individuals with sporadic MMVP identified 145 variants across 104 genes, including five previously implicated MMVP genes (*COL1A2, FLNA, FLNC, TGFB1, TTN*) in 14 patients. Notably, this study uncovered three novel MMVP-associated genes: *PRDM5* (ECM organization), *ZNF469* (collagen homeostasis, brittle cornea syndrome gene), *COL11A1* (collagen fibril formation) (220). Another targeted WES screen of 101 nonsyndromic MMVP probands (96% Barlow's disease) identified likely pathogenic variants in six cardiomyopathy-associated genes, *DSP, HCN4, MYH6, TMEM67, TRPS1, and TTN* (220). Updated GWAS, incorporating >8 million SNPs via TOPMed imputation and the UK Biobank, find additional loci emerged, including: Chr1 (*SYT2*), Chr8 (*MSRA*), Chr19 (*FBXO46*) (218). Together, these genomic studies highlight the disorder's genetic heterogeneity and the prominent role of mechano-transduction and ECM regulation in its pathogenesis.

#### 6.2.6 Epigenetics of myxomatous mitral valve prolapse

Epigenomic studies in human valves further implicate *TNS1*. Assay for transposase-accessible chromatin sequencing (ATAC-seq) performed on 11 pathogenic and 7 nonpathogenic human MVs revealed that MMVP-associated variants are enriched in valve open chromatin regions, particularly at the *IGFBP2/IGFBP5/TNS1* regulatory locus (221). The sentinel variant rs6723013 resides within an active enhancer region. CRISPR–Cas9 deletion of the sequence containing rs6723013 in human fibroblasts selectively increased TNS1 expression, confirming that this regulatory element modulates *TNS1* transcription. Chromatin conformation capture analysis further demonstrated long-range interactions linking the enhancer to the *TNS1* promoter, providing strong mechanistic evidence that this locus influences MMVP risk by altering *TNS1* regulation (221). These genetic and functional studies position *TNS1* as a key nonsyndromic MMVP susceptibility gene, acting through impaired cytoskeletal organization, altered mechanosensing, and disrupted ECM–cell adhesion dynamics.

Therefore, a substantial body of evidence from *in vitro* studies, animal models, and human MMVP specimens demonstrates that MMVP is a polygenic and multifactorial disease and supports a central pathogenic cascade initiated by TGFβ signaling, in which VECs-driven EndoMT and activation of qVICs into aVICs drive progressive ECM remodeling and the characteristic histological changes of myxomatous leaflets. However, as highlighted by genetic studies, not all identified causal genes converge on the TGFβ pathway. Several MMVP-associated genes instead implicate pathways related to mechanotransduction, cytoskeletal organization, ciliary signaling, and cell–cell adhesion, suggesting that TGFβ represents one major, but not exclusive, pathogenic axis. These observations reinforce the concept that MMVP arises from the convergence of multiple genetic and molecular pathways, with TGFβ–dependent remodeling acting as a common downstream effector in many, but not all, disease contexts. Future studies integrating genetics, epigenetics, biomechanics, and cell- or tissue-specific signaling will be required to define the full pathogenic landscape of MMVP and to identify additional therapeutic targets beyond TGFβ signaling.

## 7 Conclusions

Although RMS, CMS, and MMVP all culminate in impaired MV function, they arise from fundamentally different etiologies and follow distinct molecular and pathological trajectories. RMS is an acquired, immune-mediated disease triggered by GAS infection, leading to chronic inflammation, progressive leaflet fibrosis, commissural fusion, calcification, and marked chordal shortening. CMS, by contrast, reflects developmental malformations of the mitral leaflets, chordae, or PMs; the defect is fixed during embryogenesis and frequently occurs alongside other congenital cardiac anomalies. MMVP, particularly nonsyndromic MMVP, represents a degenerative process driven by aberrant EndoMT, VIC activation, and TGFβ–dependent ECM remodeling, resulting in leaflet redundancy, proteoglycan expansion, collagen fragmentation, and chordal elongation or rupture.

These differences are also mirrored at the genetic level. Despite extensive genomic studies, no clear monogenic causes for RMS have been identified that can account for the immune-related pathways or reflect the post-infectious, autoimmune nature of the disease. In contrast, genomic studies in MMVP have identified multiple causal or strong candidate genes, including *DCHS1, FLNA, DZIP1,* and *TNS1*, highlighting its fundamentally genetic and mechano-regulatory origin. On the other hand, CMS, due to its rarity, has far fewer molecular or genetic studies, and its underlying developmental gene networks remain largely unexplored. Notably, while large-scale genomic datasets exist for RMS and MMVP (WGS, WES, GWAS), epigenomic studies remain scarce across all three conditions, which represents a promising future direction for advancing our understanding of valve disease biology.

It is worth noting that, despite their distinct etiologies, immune-mediated (RMS), developmental (CMS), and degenerative/genetic (MMVP), these diseases converge on a shared endpoint: progressive structural failure of the MV apparatus. A particularly important, often underappreciated, unifying theme is that CT pathology plays a decisive role in clinical deterioration. In RMS and CMS, chordal fibrosis and shortening critically restrict leaflet mobility, precipitating severe stenosis; in MMVP, chordal elongation or rupture is the major trigger for acute or severe regurgitation and frequently necessitates surgical intervention. Thus, across this diverse spectrum of disease, the subvalvular apparatus, including the CTs, emerges as a central determinant of functional decline, pointing up the need for deeper basic scientific investigation into chordal biology and its remodeling across pathological contexts.

Understanding both shared mechanisms (ECM remodeling, structural degeneration) and the disease-specific pathways (immune activation in RMS, developmental disruption in CMS, mechano-genetic dysregulation in MMVP) is essential for improving diagnostic precision, tailoring therapeutics, and guiding the next generation of translational research aimed at MV disease prevention.
